# Prokaryotic Diversity in Mangrove Sediments across Southeastern China Fundamentally Differs from That in Other Biomes

**DOI:** 10.1128/mSystems.00442-19

**Published:** 2019-09-10

**Authors:** Cui-Jing Zhang, Jie Pan, Chang-Hai Duan, Yong-Ming Wang, Yang Liu, Jian Sun, Hai-Chao Zhou, Xin Song, Meng Li

**Affiliations:** a Institute for Advanced Study, Shenzhen University, Shenzhen, China; b Key Laboratory of Optoelectronic Devices and Systems of Ministry of Education and Guangdong Province, College of Optoelectronic Engineering, Shenzhen University, Shenzhen, China; c College of Life Sciences and Oceanography, Shenzhen University, Shenzhen, China; Argonne National Laboratory

**Keywords:** mangroves, biodiversity, community assembly, stochastic, deterministic, mangrove ecosystems, neutral theory, niche theory

## Abstract

Mangroves, as a blue carbon reservoir, provide an environment for a variety of microorganisms. Mangroves lie in special locations connecting coastal and estuarine areas and experience fluctuating conditions, which are expected to intensify with climate change, creating a need to better understand the relative roles of stochastic and deterministic processes in shaping microbial community assembly. Here, a study of microbial communities inhabiting mangrove sediments across southeastern China, spanning mangroves in six nature reserves, was conducted. We performed high-throughput DNA sequencing of these samples and compared them with data of 1,370 sediment samples collected from the Earth Microbiome Project (EMP) to compare the microbial diversity of mangroves with that of other biomes. Our results showed that prokaryotic alpha diversity in mangroves was significantly higher than that in other biomes and that microbial beta diversity generally clustered according to biome types. The core operational taxonomic units (OTUs) in mangroves were mostly assigned to *Gammaproteobacteria*, *Deltaproteobacteria*, *Chloroflexi*, and *Euryarchaeota*. The majority of beta nearest-taxon index values were higher than 2, indicating that community assembly in mangroves was better explained through a deterministic process than through a stochastic process. Mean annual precipitation (MAP) and total organic carbon (TOC) were main deterministic factors explaining variation in the microbial community. This study fills a gap in addressing the unique microbial diversity of mangrove ecosystems and their microbial community assembly mechanisms.

**IMPORTANCE** Understanding the underlying mechanisms of microbial community assembly patterns is a vital issue in microbial ecology. Mangroves, as an important and special ecosystem, provide a unique environment for examining the relative importance of stochastic and deterministic processes. We made the first global-scale comparison and found that microbial diversity was significantly different in mangrove sediments compared to that of other biomes. Furthermore, our results suggest that a deterministic process is more important in shaping microbial community assembly in mangroves.

## Abstract (precis)

Understanding the underlying mechanisms of microbial community assembly patterns is a vital issue in microbial ecology. Mangroves, as an important and special ecosystem, provide a unique environment for examining the relative importance of stochastic and deterministic processes. We made the first global-scale comparison and found that microbial diversity was significantly different in mangrove sediments compared to that of other biomes. Furthermore, our results suggest that a deterministic process is more important in shaping microbial community assembly in mangroves.

## INTRODUCTION

Exploring patterns of microbial community assembly has generated deep interest among ecologists. It is a fundamental question in the field of microbial ecology (1). Neutral theory and niche theory are two of the most well documented ecological theories explaining community assembly patterns (2). These theories, originally developed for use in plants and animals, have been verified for microbes in recent years (3, 4). Neutral theory assumes that stochastic processes (dispersal, local extinction, and ecological drift) cause variability in microbial community composition (5). According to this theory, microbes disperse and drift according to chance. Geographical distance may lead to a shift in the microbial community, and the similarity of a community undergoes a distance-decay relationship (6). Niche theory, however, proposes that microbial dispersal is unlimited and that microbial community assembly is primarily driven by deterministic factors (environmental selection) (7). Bass-Becking hypothesized that “everything is everywhere, but the environment selects,” due to the small size, high rates of population growth, and the vast abundance of microbes. Considering that microbial community composition is heterogeneous among various biome types (8, 9), which process is predominant for shaping the microbial community across and within biomes remains unclear (1, 10).

Mangrove forests are widely distributed in tropical and subtropical coasts, providing protection from waves and storms (11). They inhabit approximately 0.5% of the coastal area and contribute 10 to 15% of global carbon storage, which gives mangroves the distinction of being a “blue carbon reservoir” (11, 12). Mangrove sediments exhibit special environmental conditions such as high salinity and low oxygen, supporting a variety of unique microorganisms that play an important role in nutrient biogeochemical cycles, such as methane cycling, ammonia oxidation, and sulfate reduction (13, 14). Since the 1980s, the exotic salt marsh grass Spartina alterniflora has invaded the coast of China in some specific areas, resulting in a serious threat to the native mangrove ecosystems (15). Since mangroves exert important ecological functions and locate in a fluctuating environment at the land-ocean interface, they provide a unique ecosystem to test the above ecological theories. It has been reported that the microbial community in mangroves can be shaped by deterministic processes. For example, pH and sediment nutrients (organic matter and total nitrogen) are reported to be the most significant factors affecting the microbial community in mangroves (16, 17). Quantifying the relative importance of stochastic and deterministic processes will contribute to understanding the potential mechanisms of microbial community assemblage.

Recent studies have investigated the microbial communities in mangrove ecosystems (13, 17–19). However, how they are different from those of other biomes such as freshwater, ocean water, salt water lake, and hot spring sediments remains unclear. Keystone taxa in various biomes, including terrestrial, aquatic, and human microbiomes, have been reported before (20) but not in mangrove ecosystems. The rapid development of high-throughput sequencing technology provides a great quantity of microbial sequence data. The Earth Microbiome Project (EMP) has assembled a large set of samples submitted by scientists around the world and uses standard primers (i.e., 515F/806R) and protocols to provide a reference set against which new data sets can be compared (21, 22). It offers opportunities for clarifying the differences between microbial communities in mangroves and those of other biomes. Microbial databases of sediment samples in the EMP encompass a wide range of habitats, such as freshwater rivers, freshwater lakes, salt water lakes, and hot springs. However, the representation of mangrove ecosystems is currently quite limited.

In this study, we characterized the mangrove microbiome using 78 sediment samples from six mangrove nature reserves across southeastern China and compared their microbial diversity with that of other major biomes. We performed a global-scale meta-analysis of published available 16S rRNA data sets including 1,370 sediment samples from 26 studies. The data sets include six different types of biomes (freshwater river, freshwater lake, coastal zone, ocean, salt water lake, and hot spring). We hypothesized(i) that microbial diversity in mangrove ecosystems will be distinct from that other biomes, (ii) that a deterministic process prevails in shaping microbial community assembly in mangrove ecosystems, and (iii) that pH and sediment nutrients will play a vital role in driving microbial assemblage in mangrove ecosystems.

## RESULTS

### Prokaryotic diversity and the community assembly patterns in different biomes.

**FIG 1:**
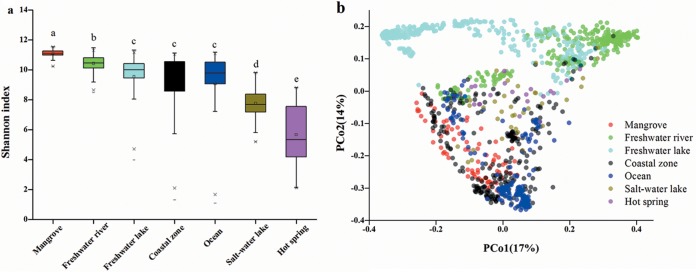
Alpha and beta diversity. (a) Alpha diversity for different biomes, with box plots showing median, interquartile range, and 1.5× the interquartile range (with outliers). The numbers of replicates used for different biomes are as follows: mangrove, 78; freshwater river, 322; freshwater lake, 408; coastal zone, 255; ocean, 181; salt water lake, 36; and hot spring, 18. Different letters indicate significant differences among different biomes (*P < *0.05, *post hoc* test after ANOVA). (b) Beta diversity in 1,298 samples. Principal coordinates analysis (PCoA) of Bray-Curtis distance matrix of different biomes is shown, according to the legend on the figure.

Overall, we analyzed a collection of 1,448 16S rRNA gene libraries from sediment samples after combining our data with the EMP data. After rarefying data to 10,000 sequences per sample, only 1,298 samples remained. Alpha diversity, as measured by the Shannon index, was significantly higher in mangroves than in the other biomes (Fig. 1a). Principle coordinates analysis (PCoA) revealed that prokaryotic beta diversity in mangrove ecosystems was similar to that in some other coastal zones but showed a distinct cluster compared with analysis of other biomes (Fig. 1b). A permutational multivariate analysis of variance (PERMANOVA) based on Bray-Curtis dissimilarity corroborated that biome types imposed a significant influence on microbial beta diversity (*P = *0.001, *R*^2^ = 0.266). The microbial community demonstrated a special composition in mangrove ecosystems, which was predominantly composed of members of *Gammaproteobacteria* (19.2%), followed by *Deltaproteobacteria* (17.9%) and *Chloroflexi* (13.8%) (see Fig. S2 in the supplemental material). The relative abundances of *Chloroflexi*, *Planctomycetes*, *Gemmatimonadetes*, *Deltaproteobacteria*, and *Epsilonproteobacteria* were significantly higher in mangrove ecosystems than in other biomes (Fig. S2) (*P < *0.05).

**FIG 2:**
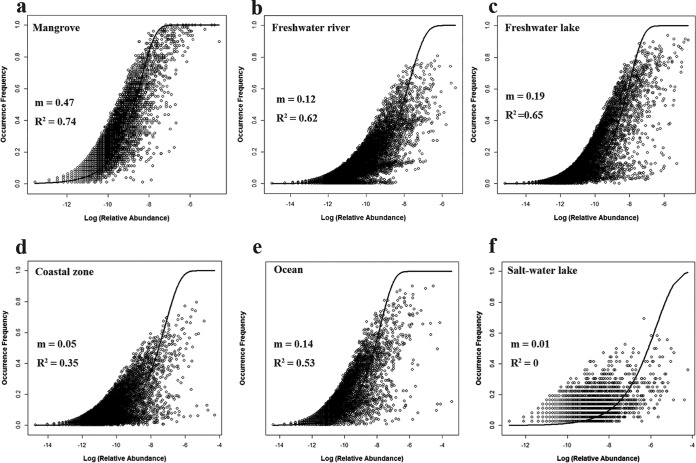
Relationships between occurrence frequency and relative abundance of OTUs in different biomes. The numbers of replicates used for different biomes, as indicated, are as follows: mangrove, 78; freshwater river, 322; freshwater lake, 408; coastal zone, 255; ocean, 181; and salt water lake, 36.

The neutral community model (NCM) fitted well to microbial community assembly in mangrove, freshwater river, and freshwater lake biomes (*R*^2^ > 0.6). However, this model did not fit well the microbial community assembly in coastal zone, ocean, salt water lake, and hot spring biomes (Fig. 2). The estimated immigration rate (*m*) in mangroves (*m* = 0.47) was higher than that in other biomes, suggesting that there were more dispersal and ecological drift in mangroves than in other biomes. Variation partition analysis (VPA) revealed that a total of 13.9% of the microbial community variations were explained by geographic factors and environmental factors (Fig. S3a). Geographic factors included latitude and longitude, and environmental factors included mean annual temperature (MAT), mean daily temperature range (MDR), mean annual precipitation (MAP), precipitation seasonality, and salinity. We found that microbial community dissimilarity increased significantly with geographical distance (Fig. S3b) (Mantel test; *r* = 0.297, *P < *0.001). In addition, we found that the correlation between microbial community dissimilarity and environmental dissimilarity was stronger (Fig. S3c) (Mantel test; *r *=* *0.476, *P < *0.001). Redundancy analysis (RDA) revealed that salinity was the most important environmental factor affecting the microbial community in sediment across the globe (Fig. S3d).

### Prokaryotic abundance and diversity in mangrove ecosystems.

**FIG 3:**
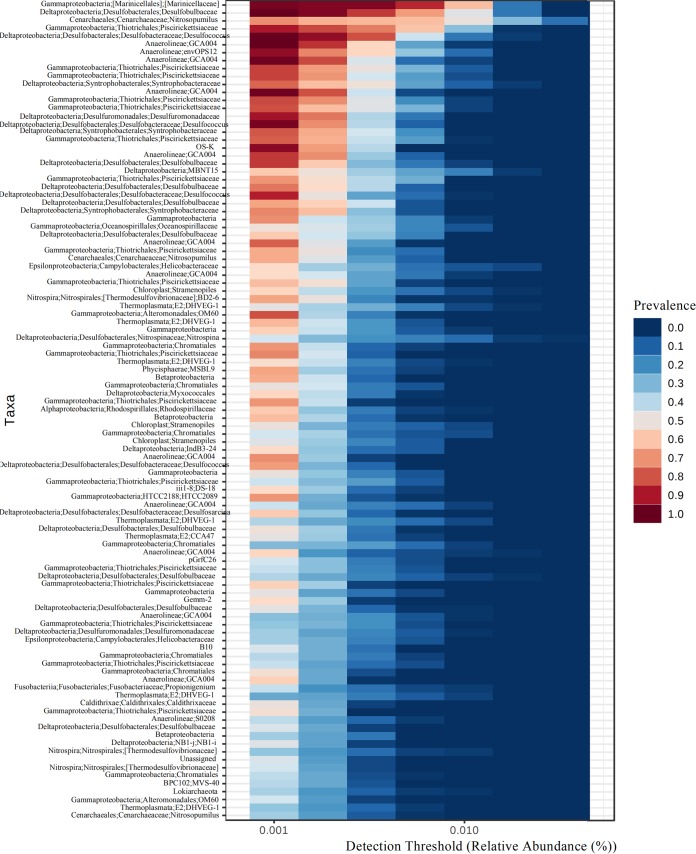
Prevalence of core microbiomes at different detection thresholds across 78 samples in mangrove ecosystems.

We found 103 core operational taxonomic units (OTUs) (i.e., prevalence in >20% of samples) in 78 sediment samples in mangrove nature reserves across southeastern China at a minimum detection threshold of 0.001% relative abundance. The observed OTUs with the highest prevalence belonged to the *Marinicellaceae*, *Desulfobulbaceae*, *Piscirickettsiaceae*, and *Desulfococcus* (Fig. 3). These OTUs were abundant and ubiquitous across sites and plant types in mangrove ecosystems. Core OTUs were mostly assigned to *Gammaproteobacteria* (32.0%), *Deltaproteobacteria* (23.3%), *Chloroflexi* (12.6%), and *Euryarchaeota* (6.8%).

**FIG 4:**
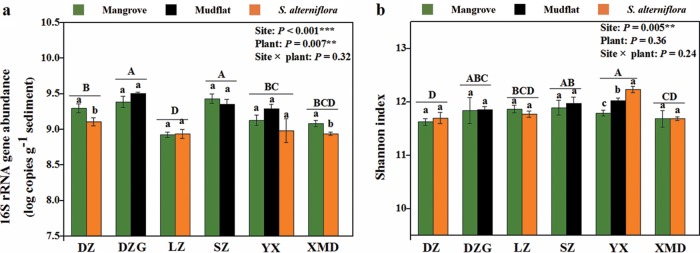
Prokaryotic abundance and alpha diversity in mangrove ecosystems. (a) Log-transformed abundances of the 16S rRNA gene across sites and plants. (b) Shannon index across sites and plants. Error bars represent standard errors (*n* = 6). The upper case letters (A, B, C, D) indicate significant differences among different sites (one-way ANOVA; Duncan test; *P* < 0.05). The lower case letters (a, b) indicate significant differences among plant types at each site (one-way ANOVA; Duncan test; *P* < 0.05).

**FIG 5:**
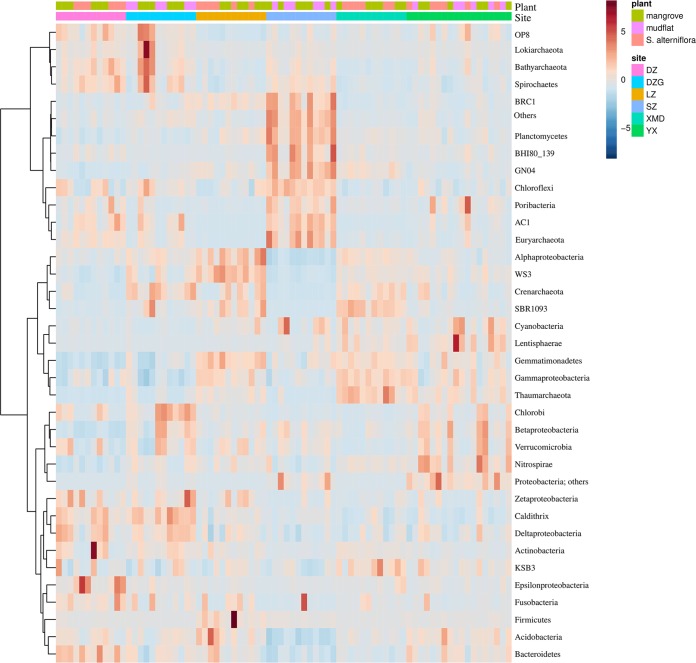
Heat map clustering analysis based on the proportion data of dominant phyla/classes in each sample.

There were significant effects of sites and plants on prokaryotic abundance (Fig. 4a) (*P* < 0.01). In particular, we found the highest microbial abundance in Dongzhaigang National Nature Reserve (DZG) and Shenzhen Futian National Nature Reserve (SZ). For Danzhou Xinyinggang Nature Reserve (DZ) and Ximendao National Marine Reserve (XMD), the abundances were significantly higher in sediments covered with mangroves than in those covered with *S. alterniflora*. Alpha diversity, as measured by the Shannon index, was only significantly different among sites but not plant types (Fig. 4b). Microbial alpha diversity was the highest in Yunxiao Zhangjiangkou National Nature Reserve (YX). PCoA and PERMANOVA based on Bray-Curtis distance revealed that prokaryotic beta diversity shifted across different sites and plant types (Fig. S4 and Table S2) (*P* = 0.001). Interestingly, our results revealed that there was a significant interaction between sites and plant types on the prokaryotic community in sediment (site-plant interaction, *P* = 0.001, *R*^2^ = 0.14). According to the data shown in Fig. S4a, the effects of plant types on the community in YX, DZG, and DZ were bigger than those in XMD, SZ, and Leizhou Nature Reserve (LZ). The heat map clustering analysis identified microbial taxa that varied significantly between sites (Fig. 5). The relative abundances of *Lokiarchaeota* and *Bathyarchaeota* were higher in DZG. The relative abundances of *Planctomycetes* and *Euryarchaeota* were higher in SZ. The relative abundances of *Thaumarchaeota* and *Nitrospirae* were higher in YX. The linear discriminant analysis effect size (LEfSe) showed the top 25 significant biomarkers that were responsible for differences between sediment types (Fig. S5). In mangrove sediments, *Bellilinea*, *Desulfatibacillum*, and *Chloroflexus* were significantly more abundant. In contrast, *Thiohalomonas* and *Pacificimonas* were overrepresented in *S. alterniflora* sediments.

### Assessing the prokaryotic community assembly patterns and driving factors in mangrove ecosystems.

**FIG 6:**
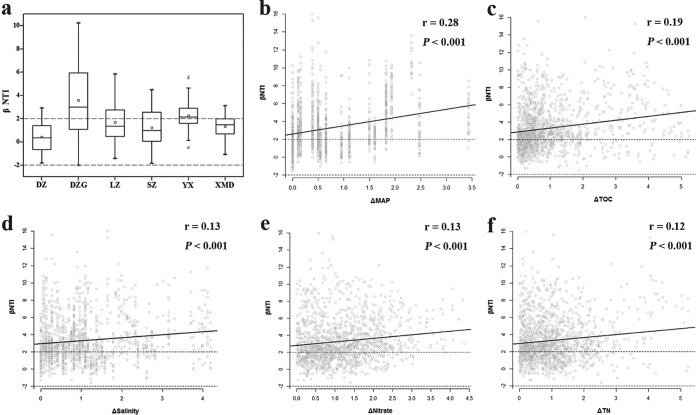
Patterns of β-nearest-taxon index (βNTI). (a) Box plots of βNTI values in mangroves across different sites showing the median, interquartile range, and 1.5× the interquartile range (with outliers). The relationships between βNTI and changes in mean annual precipitation (MAP) (b), total organic carbon (TOC) (c), salinity (d), nitrate (e), and total nitrogen (TN) (f) are plotted. Associated correlation coefficients and *P* values are provided on each panel. Horizontal dashed lines (βNTI values of 2 and −2) indicate thresholds of significance.

Since the NCM did not explain 100% of the community assembly in mangroves, we used the beta nearest-taxon index (βNTI) to explore the relative roles of stochastic and deterministic processes in shaping microbial community assembly (Fig. 6). Although the average values of βNTI in DZ, LZ, SZ, and XMD were between −2 and 2, the majority of the βNTI values (66.3%) among all the samples were higher than 2, suggesting that a deterministic process (variable selection) played a more important role in mangroves. βNTI values among all the samples were significantly correlated to changes in mean manual precipitation (MAP), total organic carbon (TOC), salinity, nitrate, and total nitrogen (TN). VPA revealed that a total of 65.3% of the community variations were explained by geographical and environmental factors (Fig. S6a). Among them, geographical factors (latitude and longitude) independently explained 3.9% of the total variances, indicating a significant but weak correlation with the dissimilarity of the microbial community (Fig. S6b) (Mantel test; *r *=* *0.176, *P < *0.001). Environmental factors (pH, salinity, MAT, MAP, ammonia, nitrite, nitrate, TOC, and TN) independently accounted for 48.9% of the total variances, showing strong correlations with microbial compositions (Fig. S6c) (Mantel test; *r *=* *0.474, *P < *0.001). Geographical and environmental factors interactively explained 12.5% of the total variances, which possibly resulted from the significant correlation of MAT with latitude. Moreover, RDA revealed that the first two axes accounted for 49.8% of the total variance, and MAP explained most of the variation in the microbial community compositions (Fig. S6d).

Correlation analyses were further performed to examine the relationships between the environmental variables and prokaryotic abundance, alpha diversity, and beta diversity (Table 1). Microbial abundance showed a significant and positive relationship with MAP, ammonia, TOC, and TN. Significantly negative correlations between relative abundances of *Betaproteobacteria*, *Deltaproteobacteria,* and *Verrucomicrobia* and pH and between relative abundances of *Planctomycetes* and *Thaumarchaeota* and salinity were found (Table 1) (*P < *0.05). Moreover, relative abundances of *Gammaproteobacteria*, *Planctomycetes*, and *Thaumarchaeota* were negatively related to MAT. *Betaproteobacteria* and *Chloroflexi* relative abundances were positively related to MAP, while the opposite pattern was observed for relative abundances of *Alphaproteobacteria*, *Gammaproteobacteria*, *Acidobacteria*, and WS3 (Table 1) (*P < *0.05). Finally, nitrite was negatively related to the relative abundance of *Deltaproteobacteria* and positively related to relative abundances of *Planctomycetes* and *Thaumarchaeota*, while nitrate was negatively related to the relative abundance of *Chloroflexi*. TOC was significantly and positively related to the relative abundances of *Chloroflexi* but negatively related to those of *Gammaproteobacteria*, *Thaumarchaeota*, and *Cyanobacteria*. TN had a positive effect on *Chloroflexi* relative abundance but a negative effect on *Alphaproteobacteria*, *Gammaproteobacteria*, and *Gemmatimonadetes* (Table 1) (*P < *0.05).

**TABLE 1:** Correlation coefficients (Spearman’s rho) between prokaryotic abundance, diversity, and composition with environmental factors^*a*^

Parameter or phylum	Correlation coefficient (ρ)								
	pH	Salinity	MAT	MAP	Ammonia	Nitrite	Nitrate	TOC	TN
Microbial abundance	−0.25	0.25	0.37	0.45*	0.53***	−0.22	−0.16	0.56***	0.45*
Alpha diversity (Shannon index)	−0.17	−0.21	−0.1	0.39	0.1	0.23	0.11	−0.1	−0.01
*Alphaproteobacteria*	−0.2	−0.03	−0.12	−0.49***	0	0.04	0.18	−0.17	−0.46*
*Betaproteobacteria*	−0.62***	−0.39	−0.13	0.66***	0.25	0.23	−0.16	0.21	0.17
*Deltaproteobacteria*	−0.52***	0.34	0.31	−0.24	0.18	−0.43*	0.1	0.42	0.07
*Epsilonproteobacteria*	0.18	0.32	0.27	−0.21	0.04	−0.39	−0.01	0.05	−0.08
*Gammaproteobacteria*	0.22	−0.33	−0.46*	−0.43*	−0.25	0.22	0.33	−0.62***	−0.48***
*Chloroflexi*	−0.35	0.18	0.32	0.5***	0.25	−0.06	−0.44*	0.64***	0.5***
*Bacteroidetes*	−0.28	0.1	0.1	−0.18	0.19	−0.29	0.38	−0.01	−0.02
*Planctomycetes*	0.34	−0.43*	−0.45*	0.13	−0.21	0.53***	−0.1	−0.39	−0.08
*Acidobacteria*	−0.26	−0.23	−0.27	−0.44*	−0.18	0.11	0.26	−0.25	−0.33
*Euryarchaeota*	0.4	0.21	0.2	0.27	−0.05	−0.04	−0.16	0.15	0.33
*Gemmatimonadetes*	−0.03	−0.29	−0.37	−0.39	−0.14	0.32	0.12	−0.38	−0.5***
*Bathyarchaeota*	0.25	0.3	0.39	0.21	−0.05	−0.26	−0.08	0.14	0.15
*Nitrospirae*	−0.27	−0.41	−0.36	0.03	−0.33	0.17	0.06	−0.15	−0.16
*Thaumarchaeota*	0.05	−0.59***	−0.67***	−0.2	−0.37	0.44*	0.31	−0.57***	−0.36
WS3	−0.36	0.03	0	−0.45*	0.02	−0.08	0.11	−0.03	−0.34
*Verrucomicrobia*	−0.64***	−0.4	−0.23	0.3	0.11	0.07	0.26	0.01	0.08
*Cyanobacteria*	0.05	−0.38	−0.41	−0.02	−0.12	0.36	0.3	−0.47***	−0.2

a MAT, mean annual temperature; MAP, mean annual precipitation; TOC, total organic carbon; TN, total nitrogen. *, *P *<* *0.05; **, *P *<* *0.01; ***, *P *<* *0.001.

## DISCUSSION

Our study characterized the mangrove microbiome and identified the prevailing process of community assemblage and major environmental drivers. Microbial alpha diversity was significantly higher in mangrove ecosystems than in other biomes, and microbial beta diversity in mangroves also differed significantly from that of other biomes. In addition, we found that deterministic processes prevailed in explaining the variation in microbial beta diversity in mangrove ecosystems, suggesting that the niche theory was more important for microbial community assembly. Furthermore, our findings revealed that MAP and TOC were the most significant environmental factors related to the microbial community in mangrove ecosystems.

An important conclusion drawn from our large-scale analysis of the results of 1,448 sediment samples around the globe was that microbial alpha diversity was significantly higher in mangroves than in other biomes. This finding is in agreement with previous results (14, 17, 23). Relatively high temperature and nutrient availability in mangrove sediments may explain the observed higher diversity. It might also be because mangroves are located in a buffer zone connecting land and ocean (24). River water discharges nutrients from upstream to the mangrove sediments (12). In addition, it was observed that microbial composition showed distinctive patterns among different biomes. *Firmicutes* have been suggested to be able to form endospores to adapt to detrimental conditions (25), giving this group an advantage in salt water lakes. *Chloroflexi*, *Planctomycetes*, and *Deltaproteobacteria* were found to be significantly more abundant in mangrove ecosystems than in other biomes (see Fig. S2 in the supplemental material). The metabolic versatility of *Chloroflexi* and *Deltaproteobacteria* can provide a competitive advantage for surviving in fluctuating environments (26, 27). It has been reported that *Deltaproteobacteria* were associated with higher salinity (28). The relative abundance of *Planctomycetes* increased with increasing C content in soil (29). The high salinity and high concentrations of organic carbon, in part, explain the observed association of these bacteria in mangrove ecosystems.

We selected abundant and ubiquitous OTUs existing in 78 sediment samples and explored their prevalence to reveal spatially independent and core microbiomes in mangrove ecosystems (30). Our analyses showed that the top 103 core OTUs were mostly assigned to *Gammaproteobacteria*, *Deltaproteobacteria*, *Chloroflexi*, and *Euryarchaeota* (Fig. 3). The observed OTUs with highest prevalence belonged to *Marinicellaceae*, *Desulfobulbaceae*, *Nitrosopumilus*, *Piscirickettsiaceae*, and *Desulfococcus*. *Desulfobulbaceae* and *Desulfococcus* in the class *Deltaproteobacteria* are anaerobic and sulfate-reducing bacteria (SRB) (31, 32). *Nitrosopumilus* in the phylum *Thaumarchaeota* is a chemoautotrophic ammonia-oxidizing archaea living in an aerobic environment (33). *Marinicellaceae* and *Piscirickettsiaceae* in the class *Gammaproteobacteria* are aerobes found in water. *Euryarchaeota* have been already reported to be distributed widely in estuarine sediments (34). In our research, core OTUs in the phylum *Euryarchaeota* all fell into the class *Thermoplasmata*. The prevalence of *Deltaproteobacteria*, *Thaumarchaeota*, and *Euryarchaeota* in mangrove ecosystems suggests a potential for sulfate reduction, ammonia oxidation, and organic matter decomposition (35, 36).

In mangrove ecosystems across southeastern China, we examined microbial abundance and diversity in different sediment types and sites and how they corresponded to environmental factors. Our study provided strong evidence that sediment types significantly affected microbial abundance and beta diversity, as previously observed with distinct microbial communities under tree species (37–39). The relative abundances of *Bellilinea*, *Desulfatibacillum*, and *Chloroflexus* were higher in mangrove sediments. *Bellilinea* and *Chloroflexus* in the phylum *Chloroflexi* have been reported to be capable of anaerobic degradation of organic compounds (40). *Desulfatibacillum*, known as a sulfate-reducing bacterium, is abundant in coastal surface sediments (41). In contrast, the relative abundances of *Thiohalomonas* and *Pacificimonas* were higher in *S. alterniflora* sediments. *Thiohalomonas* (known as a purple sulfur bacterium and N_2_ fixer) imply a potential for carbon and nitrogen fixation in this sediment type (42). *Pacificimonas* is considered to be an oligotroph (43), indicating that invasion of *S. alterniflora* might reduce nutrient availability in sediments. On the other hand, microbial abundance and diversity were also found to be different among mangrove sites. Abundance was observed to be high in SZ and DZG. This phenomenon can be attributed, at least in part, to high nutrient availability in these areas (24, 44). Significantly positive correlations between microbial abundance and MAP, ammonia, TOC, and TN in our study strongly supported the above demonstration. Alpha diversity was observed to be high in YX. However, there was no significant correlation between alpha diversity and environmental factors. The relative abundances of *Lokiarchaeota* and *Bathyarchaeota* were higher in DZG, which has been reported to be positively correlated with organic carbon (35, 45). The relative abundances of *Planctomycetes* and *Euryarchaeota* were higher in SZ, and the positive correlations between *Planctomycete*s and nitrite and between *Euryarchaeota* and TOC implied a potential for anaerobic ammonia oxidation and methane metabolism in mangroves in this area (46, 47). The relative abundances of *Thaumarchaeota* and *Nitrospirae* were higher in YX, suggesting the potential for a nitrifying process in this area (48).

Furthermore, our study elucidated the relative importance of stochastic and deterministic processes to microbial community assembly in mangroves. Stochastic processes played an important role in mangroves compared with the role in other biomes. This may be explained by the location of mangroves in the fluctuating environment at the land-ocean interface. There are invasions of various marine microbes (that is, high immigration rates) (49, 65). The average values of βNTI in DZ, LZ, SZ, and XMD were between −2 and 2, indicating that microbial community assembly was dominated by stochastic processes (dispersal and ecological drift of diverse marine microbes) in these areas at the local scale. However, putting all of the 78 samples together, most of βNTI values (66.3%) were >2, indicating that a deterministic process was more important in mangroves across southeast China. The possible reason might be that the environmental conditions in different mangrove sites leads to a difference in microbial community. The local environmental conditions (climate, pH, salinity, and nutrients availability) may be more important than marine input in driving microbial community assembly on a regional scale. VPA provided strong evidence that environmental conditions had a stronger influence on microbial diversity and composition than geographical distance, as previously observed for the microbial community in paddy soil and soil of the eastern Tibetan plateau (50, 51). As complementary evidence, we found a significant, nonetheless weak, relationship between microbial community dissimilarity and geographical distances (Fig. S6b), indicating that community dissimilarity slightly increased when communities were increasingly distant in space. Neutral processes driven by geographical distance could explain some of the variation in microbial diversity (52). In contrast, community dissimilarity showed a stronger correlation with environmental dissimilarity (Fig. S6c), indicating that heterogeneous selection by environmental factors may override the effect of the neutral theory (53). Remarkably, these results suggest that microorganisms are partitioned to specialized niches (54). Thus, niche theory may be suitable for explaining biogeographic patterns of microbes at the regional scale.

The relationships between βNTI values and changes of environmental factors (pH, salinity, MAT, MAP, ammonia, nitrite, nitrate, TOC, and TN) were used to evaluate the most important deterministic factors in driving variation in the prokaryotic community in mangrove ecosystems. The microbial community composition was found to be primarily related to MAP and TOC but less so to salinity, nitrate, and TN and not significantly related to pH, MAT, ammonia, and nitrite, indicating that MAP and TOC had stronger influences on the microbial population distribution in mangrove ecosystems. Our results support previous studies highlighting the importance of factors such as climate (55) and nutrients (28). We have also found significant relationships between environmental factors and relative abundances of dominant phyla, suggesting that the presence of niche-specific phyla was influenced by environmental factors (56). As such, our work promotes understanding of the driving factors affecting the microbial community in mangrove ecosystems.

In conclusion, this study provides strong evidence that prokaryotic diversity in mangrove ecosystems across southeastern China is fundamentally different from that found in other biomes. Core OTUs in mangrove ecosystems were mostly assigned to *Gammaproteobacteria*, *Deltaproteobacteria*, *Chloroflexi*, and *Euryarchaeota*. In mangrove ecosystems, sites and sediment plants significantly influenced prokaryotic abundance and diversity. Finally, while stochastic processes explained part of the variation in the microbial community, a deterministic process was more important in determining the community assembly patterns in mangroves. Variations of prokaryotic community were strongly linked to MAP and TOC. Thus, our findings together provided comprehensive insight into the microbial community assembly in mangrove ecosystems.

## MATERIALS AND METHODS

### Study sites and sediment sampling.

There are about 17,800 ha of mangroves in China, from Zhejiang to Hainan province. Six representative mangrove nature reserves (Ximendao National Marine Reserve, XMD; Yunxiao Zhangjiangkou National Nature Reserve, YX; Shenzhen Futian National Nature Reserve, SZ; Leizhou Nature Reserve, LZ; Dongzhaigang National Nature Reserve, DZG; Danzhou Xinyinggang Nature Reserve, DZ) were selected along latitude gradients in this study (see Fig. S1 in the supplemental material). The geographical locations of the six nature reserves are significantly different. XMD represents the most northern boundary where mangroves can survive. YX represents the most northern national mangrove reserve. SZ is the only national nature reserve located in the urban hinterland. LZ is located in the south end of the mainland of China. DZG is the first Chinese mangrove wetlands included in the List of Wetlands of International Importance. DZ is located in the west coast of Hainan province. The latitudes and longitudes of the sites were recorded using a GPS unit. Based on long-term meteorological data, the mean annual temperature (MAT) and the mean annual precipitation (MAP) are listed in Table S1. Since Kandelia candel is the most common mangrove plant in coast of southeastern China, we collected mangrove sediments near the roots of Kandelia candel plants except in DZ. We collected mangrove sediments near the roots of Rhizophora stylosa plants in DZ where there were no Kandelia candel mangroves. Due to ecological invasion, there are S. *alterniflora* grasses in XMD, YX, LZ, and DZ. Basically, three types of sediments, including mangrove sediments, *S. alterniflora* sediments, and mudflat sediments were collected. Specifically, we collected samples from 13 sediments in total (at XMD, mangrove and S. *alterniflora* sediments; YX, mangrove, S. *alterniflora*, and mudflat sediments; SZ, mangrove and mudflat sediments; LZ, mangrove and S. *alterniflora* sediments; DZG, mangrove and mudflat sediments; DZ, mangrove and S. *alterniflora* sediments).

10.1128/mSystems.00442-19.1 FIG S1 Locations of the sites included in this study. The sites of mangrove sediments across southeastern China are in red (78 samples), while the sites where we downloaded data are in black (1370 samples). XMD, Ximendao; YX, Yunxiao; SZ, Shenzhen; LZ, Leizhou; DZG, Dongzhaigang; DZ, Danzhou. Download FIG S1, TIF file, 0.3 MB. Copyright © 2019 Zhang et al. 2019 Zhang et al. This content is distributed under the terms of the Creative Commons Attribution 4.0 International license.

10.1128/mSystems.00442-19.2 FIG S2 Prokaryotic composition across different biomes and effects of biomes on the relative abundances of a few microbial phyla/classes. One-way ANOVA was used to test the effects of biomes. Only the phyla/classes that are significantly higher in mangrove ecosystems are shown in the figure. Download FIG S2, TIF file, 2.7 MB. Copyright © 2019 Zhang et al. 2019 Zhang et al. This content is distributed under the terms of the Creative Commons Attribution 4.0 International license.

10.1128/mSystems.00442-19.3 FIG S3 Association between microbial community, geographical distance, and environmental drivers at a global scale. (a) Variation partition analysis that partitions relative contributions of geographical factors and environmental factors to microbial community structure. (b) Relationship between microbial community dissimilarity (Bray-Curtis distance) and geographical distance. The solid line represents the fitted linear regressions (*n* = 1,298). (c) Relationship between microbial community dissimilarity (Bray-Curtis distance) and environmental dissimilarity (Euclidean distance). The solid line represents the fitted linear regressions (*n* = 1,298). (d) Redundancy analysis (RDA) of microbial communities with environmental factors. Environmental explanatory variables were forwarded selected based on the significant test performed by the Monte Carlo permutation test (*P* < 0.05). Symbols in different colors indicate samples from different biomes. The values of RDA1 and -2 are percentages that the corresponding axis can explain. Download FIG S3, TIF file, 1.7 MB. Copyright © 2019 Zhang et al. 2019 Zhang et al. This content is distributed under the terms of the Creative Commons Attribution 4.0 International license.

10.1128/mSystems.00442-19.4 FIG S4 Prokaryotic β-diversity and taxonomic distribution in mangrove ecosystems. (a) Principal coordinates analysis (PCoA) derived from the Bray-Curtis dissimilarity matrices across different sites and plants. (b) Prokaryotic community composition shown as relative abundances of dominant phyla/classes. Less abundant phyla (total average < 1%) and unclassified microbes are collapsed into the “Others” bar. Download FIG S4, PDF file, 1.3 MB. Copyright © 2019 Zhang et al. 2019 Zhang et al. This content is distributed under the terms of the Creative Commons Attribution 4.0 International license.

10.1128/mSystems.00442-19.5 FIG S5 Microbial taxa significantly differentiated among three sediments identified by linear discriminant analysis coupled with effect size (LEfSe). The bar graph shows the LDA scores of the top 25 significant OTUs. Download FIG S5, TIF file, 2.4 MB. Copyright © 2019 Zhang et al. 2019 Zhang et al. This content is distributed under the terms of the Creative Commons Attribution 4.0 International license.

10.1128/mSystems.00442-19.6 FIG S6 Association between microbial community, geographical distance, and environmental drivers in mangrove ecosystems at a regional scale. (a) Variation portioning analysis that partitions the relative contribution of geographical factors and environmental factors on microbial community structure. (b) Relationship between microbial community dissimilarity (Bray-Curtis distance) and geographical distance. The solid line represents the fitted linear regressions (*n* = 78). (c) Relationship between microbial community dissimilarity (Bray-Curtis distance) and environmental dissimilarity (Euclidean distance). The solid line represents the fitted linear regressions (*n* = 78). (d) RDA of microbial communities with environmental factors. Environmental explanatory variables were forwarded selected based on the significant test performed by the Monte Carlo permutation test (*P* < 0.05). Symbols in different colors indicate samples from different sites. The values of RDA1 and -2 are percentages that the corresponding axis can explain. Download FIG S6, TIF file, 1.0 MB. Copyright © 2019 Zhang et al. 2019 Zhang et al. This content is distributed under the terms of the Creative Commons Attribution 4.0 International license.

10.1128/mSystems.00442-19.7 TABLE S1 Metadata of samples analyzed in this study. Download Table S1, XLSX file, 0.2 MB. Copyright © 2019 Zhang et al. 2019 Zhang et al. This content is distributed under the terms of the Creative Commons Attribution 4.0 International license.

10.1128/mSystems.00442-19.8 TABLE S2 Summary of results (*F* value and *P* value) of analysis of variance (ANOVA) showing the effects of site and plant type on dominant archaeal and bacterial phyla/classes. Values shown in bold are probability with significant results (*P < *0.05). Download Table S2, DOCX file, 0.08 MB. Copyright © 2019 Zhang et al. 2019 Zhang et al. This content is distributed under the terms of the Creative Commons Attribution 4.0 International license.

Sediment samples were collected using a stainless steel sampler (10 cm by 10 cm) in October to December 2017. At each sampling site, two depths were sampled corresponding to the surface (0 to 10 cm) and subsurface (10 to 20 cm). For each sediment type, three replicates were sampled, resulting in a total of 78 sediment samples (3 replicates × 2 depths × 13 sediment types). All samples were transferred on ice to the laboratory in 3 days. Sediment samples were separated into two sets. One sample set was stored at –40°C before DNA extraction, and the other set was stored at 4°C before physicochemical analyses.

### Environmental parameter analysis.

Salinity and pH were measured using fresh sediments, and nutrients were measured using air-dried sediments. Salinity was measured by an automatic compensation salinity refractometer (ATAGO Co., Japan). Sediment pH was determined by a pH meter (Mettler-Toledo Instruments Co., China). Sediments were air dried for a few days until the weight remained unchanged. Each sample was thoroughly mixed after passage through a 2-mm-pore-size sieve to remove roots and stones. Sediment ammonium, nitrite, and nitrate were extracted from air-dried sediments (10 g) with 100 ml of 1 M KCl by shaking at 180 rpm for 60 min. The filtered solution was determined by a continuous segmented flow analyzer (SEAL AutoAnalyzer 3 HR; Maquon, WI, USA). Sediments for total organic carbon (TOC) and total nitrogen (TN) measurements were ground to a fine powder (mesh number 100) using a mortar and pestle. Briefly, 2 g of mixed catalyst (K_2_SO_4_:CuSO4:Se at 100:10:1, passed through a no. 80 sieve) and 5 ml of concentrated H_2_SO_4_ were added into dried sediment (0.5 g) and then boiled at 150°C for 60 min and at 250°C for 240 min. Volume was fixed to 100 ml, and the concentration of the upper solution was determined using a TOC analyzer (Shimadzu, Japan).

### Molecular analysis.

Sediment genomic DNA was extracted from 0.3 g of the samples using a DNeasy PowerSoil kit (Qiagen, Germany) according to the manufacturer’s instructions. The quantity and quality of the extracted DNA were examined using NanoDrop ND-2000c UV-Vis spectrophotometer (NanoDrop Technologies, Wilmington, DE, USA). The DNA samples were stored at –20°C and used for later molecular analysis. Abundance of the prokaryotic 16S rRNA genes was quantified by quantitative PCR (qPCR) on an iCycler iQ 5 thermocycler (Bio-Rad, USA) using the primer pairs 515F (5′-GTGCCAGCMGCCGCGGTAA-3′) and 806R (5′-GGACTACHVGGGTWTCTAAT-3′) (22). The 25-μl reaction mixture contained 12.5 μl of 2× SYBR Premix Ex Taq (TaKaRa Biotechnology, Japan), 0.5 μl of each primer (10 μM), and 2 μl of diluted DNA template (1 to 10 ng). Amplification conditions were as follows: 94°C for 3 min and then 30 cycles of 45 s at 94°C, 60 s at 58°C, and 60 s at 72°C, followed by a melt curve from 58°C to 94°C at a 0.5°C increment. Standard curves were developed using 10-fold serial dilutions of plasmid containing a correct insert of the 16S rRNA gene. PCR efficiency for different assays ranged between 90% and 100% and an *R*^2^ of 0.99.

For amplicon sequencing, prokaryotic (bacteria and archaea) 16S rRNA gene fragments were amplified using the primer pairs 515F/806R (22), which were also used in Earth Microbiome Project (EMP). Amplicons were sequenced on a MiSeq platform (Illumina, San Diego, CA, USA) at Genewiz in Suzhou, China. Across the 78 samples examined, the high-throughput sequencing yielded 8,852,269 16S rRNA gene sequences in total, and the minimum sequence number for an individual sample was 90,324.

### Data collection from other studies.

For comparisons with other biomes, we collected next-generation sequencing data on sediment prokaryotic diversity using 515F and 806R primers (22) from published data and EMP data sets (21). A search using the keyword combination “sediment” and “Illumina” and the combination “mangrove” and “Illumina” was done on SCOPUS during April 2018. We collected approximately 120 references. Within these references, 26 studies were used (Table S1) in the following analyses since they met the following criteria: (i) they contained information of the spatial location (latitude and longitude) and biome types; (ii) they provided 16S rRNA gene sequences using primers 515F and 806R. A total of 1,370 sediment samples from 243 sites were found (Fig. S1). For each sediment sample included in the database, the following information was included (Table S1): (i) geographical coordinates (latitude and longitude); (ii) biome type (freshwater river, freshwater lake, coastal zone, ocean, salt water lake, and hot spring, where coastal zone is defined as area from 50-m depth nearshore ocean to 100 km inland according to the Millennium Ecosystem Assessment); (iii) climate (mean annual temperature [MAT], mean diurnal temperature range [MDR], mean annual precipitation [MAP], and precipitation seasonality). Climate data were obtained from the WorldClim database (http://www.worldclim.org). Sequences were downloaded from the European Bioinformatics Institute (EMBL-EBI) according to the accession number.

### Data processing.

Data collected from each study was combined with our sequencing data, resulting in 1,448 sediment samples from 249 sites (Fig. S1). Raw data were quality filtered to remove low-quality bases using Sickle (https://github.com/najoshi/sickle). Sequences were then processed using the Quantitative Insights into Microbial Ecology (QIIME) pipeline (57) to join the paired ends. After the removal of chimeras, UCLUST was used to pick open reference operational taxonomic units (OTUs) at 97% sequence identity (58). Representative sequences of each OTU were then aligned using PyNAST (59) and assigned based on the SILVA132 database (60). All the biological observation matrix (BIOM) files of each data set were merged using QIIME. The resultant OTU abundance tables were rarefied to an even number (10,000) of sequences per sample to ensure equal sampling depth, resulting in 1,298 samples included for the following analysis. Shannon’s index is considered an estimator of microbial alpha diversity as it has been widely used and can be compared with results of other studies.

### Statistical analysis.

**(i) Exploring microbial community assembly patterns at a global scale.** Statistical analysis of microbial community assembly patterns on a global scale was done using the 1,298 sediment samples. Analyses of variance (ANOVA) were conducted to compare the prokaryotic alpha diversity and relative abundances of major phyla/classes across different biomes. Principal coordinate analysis (PCoA) and permutational multivariate analysis of variance (PERMANOVA) based on the Bray-Curtis dissimilarity matrices on the genus level were completed to test whether prokaryotic community compositions shifted among different biomes. The neutral community model (NCM) was used to evaluate the potential role of stochastic processes in shaping microbial community assembly in different biomes (52). The relationship between occurrence frequency and relative abundance of OTUs was fitted to the model using the sncm.fit_function.r code written by Burns et al. (61).

Redundancy analysis (RDA) was used to explore effect of environmental factors on the microbial community. RDA-based variation partition analysis (VPA) was performed to determine the relative proportions of microbial community variations that can be explained by geographical factors (latitude and longitude) and environmental conditions (MAT, MDR, MAP, precipitation seasonality, and salinity). In addition, we assessed dissimilarity between microbial communities as geographical and environmental distances increased. We calculated the Bray-Curtis dissimilarity index for microbial community, Euclidean distance for geographical distances, and Euclidean distances for environmental dissimilarity (i.e., standardized Euclidean distances for different measurements among MAT, MDR, MAP, precipitation seasonality, and salinity). Significance of the associations was assessed using Mantel tests with Pearson’s correlation coefficient and 999 permutations. All statistical analyses were performed using R, version 3.3.2 (R Development Core Team, Vienna, Austria).

**(ii) Exploring microbial diversity and evaluating major drivers in mangrove ecosystems at a regional scale.** Statistical analysis of microbial diversity on a regional scale was done using the 78 sediment samples of mangrove ecosystems. To explore the core microbiome, abundant and ubiquitous OTUs (with abundances of >0.001% to total OTUs and occurrence in more than 20% of samples) were selected (62). The 16S rRNA gene copy numbers were log transformed prior to statistical analysis to satisfy the normality assumptions. ANOVA were conducted to compare the prokaryotic abundance, alpha diversity, and relative abundances of major phyla/classes across different sites and plant types. PCoA and PERMANOVA based on the Bray-Curtis dissimilarity matrices were completed to visualize shifts in the prokaryotic community compositions based on the genus level across different treatments. The distribution pattern of each phylum based on relative abundance was visualized on a heat map. Microbial taxonomies that varied significantly among the three plant types were explored using the linear discriminant analysis effect size (LEfSe) with a Kruskal-Wallis test and an α value of 0.05. Core microbiome, heat map clustering analysis, and LEfSe were performed with bioinformatics tools implemented in MicrobiomeAnalyst (63). The beta nearest-taxon index (βNTI) was used to quantify the relative importance of stochastic and deterministic processes, with rare OTUs (relative abundances of less than 0.01% of the total number of sequences) being deleted using the ses.comdistnt function (abundance.weighted = TRUE) in the MicEco R package. The dominance of a deterministic process (homogeneous and heterogeneous selection) could be inferred when βNTI values were less than −2 or greater than 2. βNTI values between −2 and 2 suggest that stochastic processes (dispersal and drift) may be more important (64). The relationships between βNTI and Euclidean distances in environmental factors were evaluated using Mantel tests with Pearson’s correlation coefficient and 999 permutations.

As described in the paragraph above on statistical analysis performed on a global scale, RDA, VPA, the distance-decay analysis, and linkage between microbial community and environmental factors were done for 78 samples of mangrove ecosystems. Here, environmental factors included pH, salinity, climate (MAT and MAP), and nutrients (ammonia, nitrite, nitrate, TOC, and TN). Relationships between microbial community composition (microbial abundance, diversity, and relative abundance of microbial phyla) and the environmental factors of pH, salinity, climate, and nutrients were explored using Spearman’s rank-order correlations. All statistical analyses were performed using R, version 3.3.2 (R Development Core Team, Vienna, Austria).

### Data availability.

The data sets supporting the conclusions of this article were deposited in the NCBI GenBank under BioProject ID PRJNA475455 and under SRA accession number SRP150213.
