# Supplementary figures and images for: Prokaryotic Diversity in Mangrove Sediments across Southeastern China Fundamentally Differs from That in Other Biomes
Source: mSystems. 2019 Sep 10;4(5):e00442-19. doi: 10.1128/mSystems.00442-19 (PMC6739103; doi:10.1128/mSystems.00442-19)

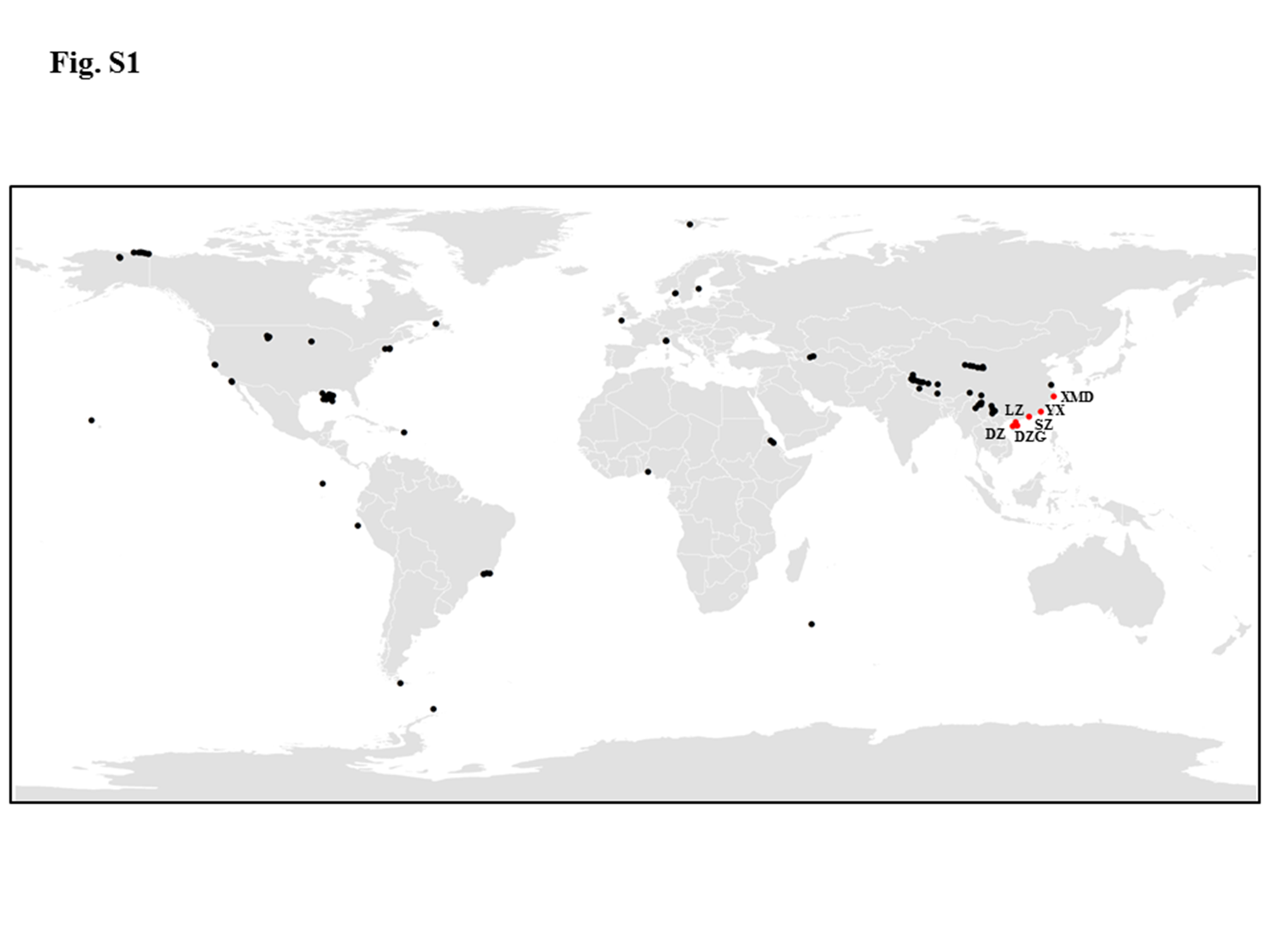

Supplement: FIG S1 [file mSystems.00442-19-sf001.tif]

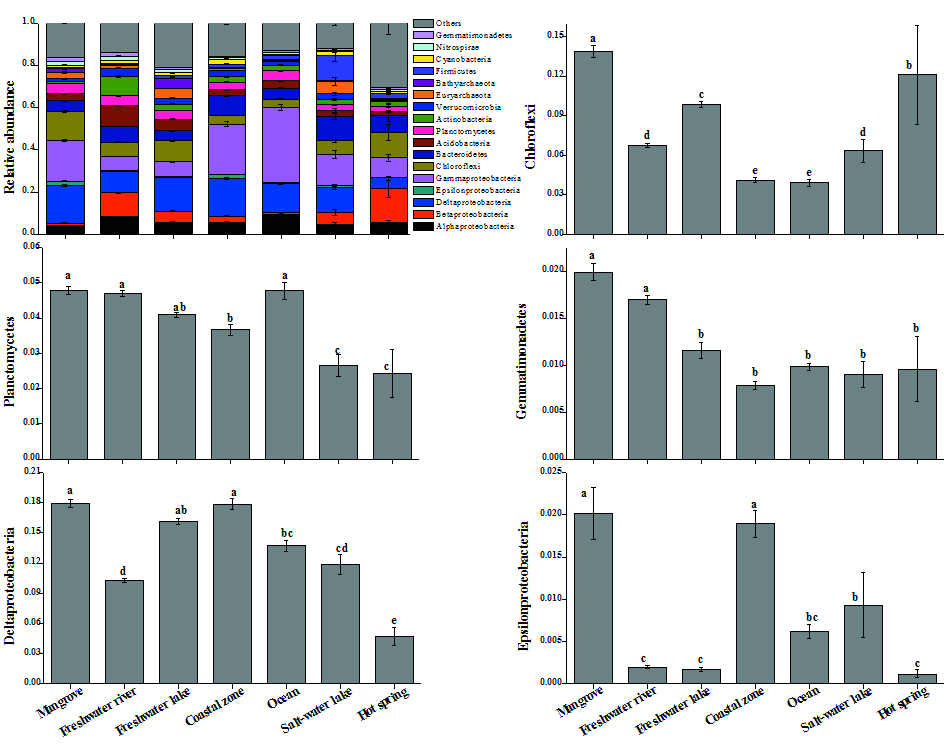

Supplement: FIG S2 [file mSystems.00442-19-sf002.tif]

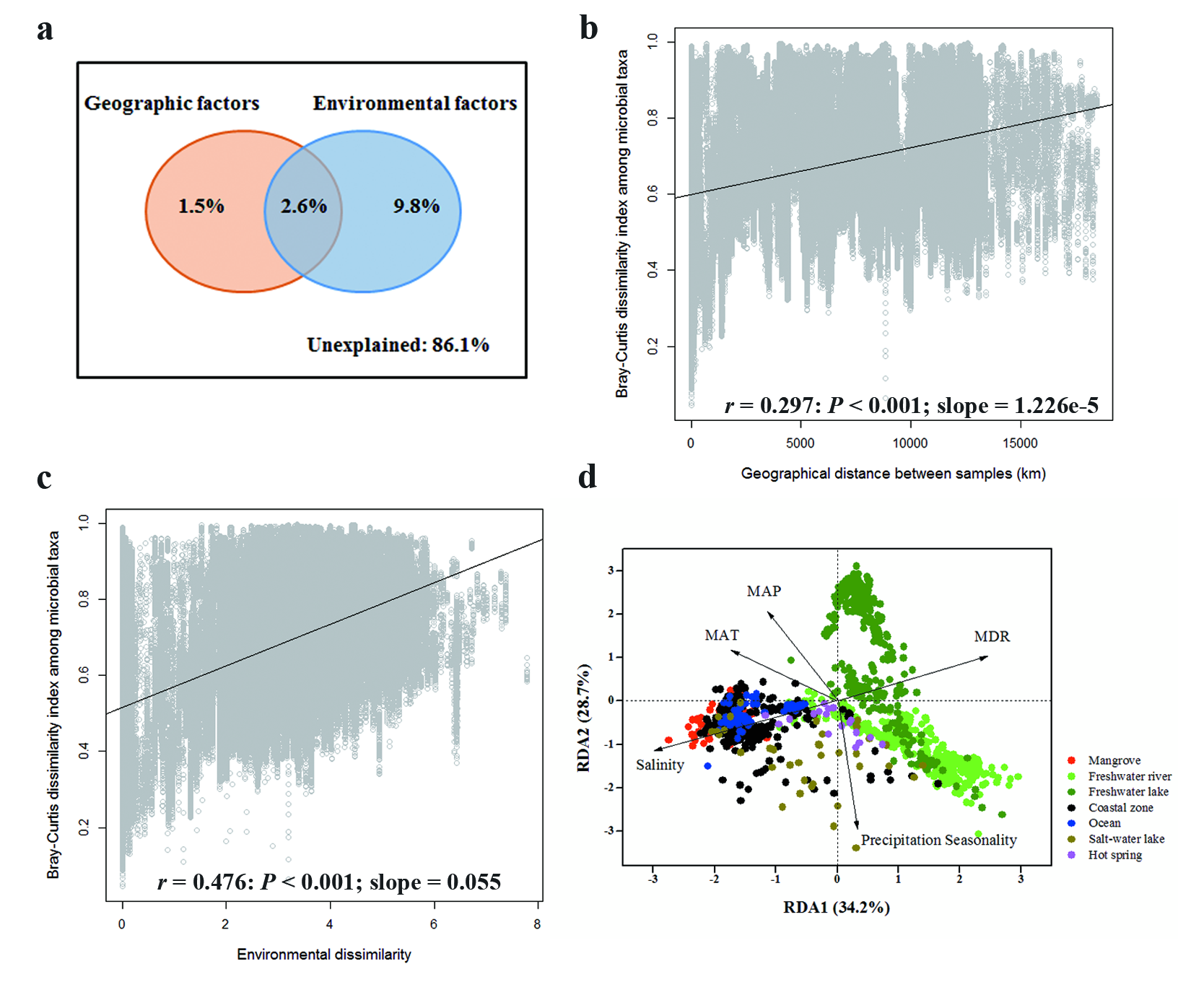

Supplement: FIG S3 [file mSystems.00442-19-sf003.tif]

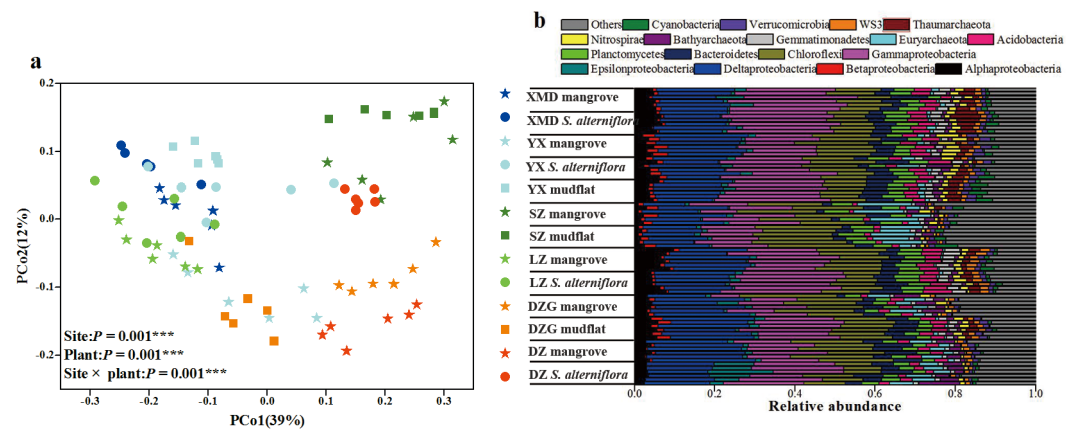

Supplement: FIG S4 [file mSystems.00442-19-sf004.pdf]

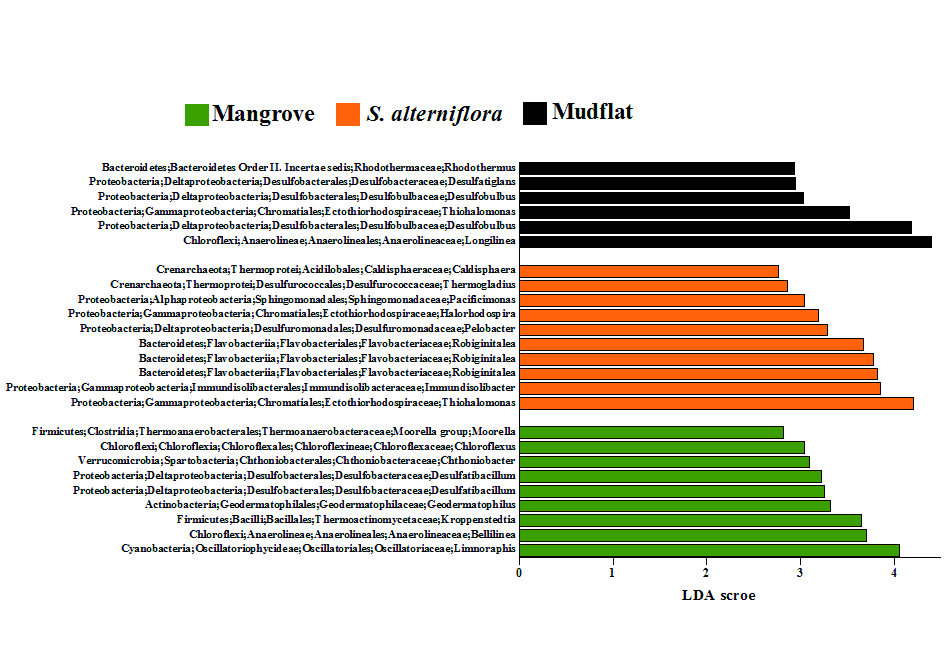

Supplement: FIG S5 [file mSystems.00442-19-sf005.tif]

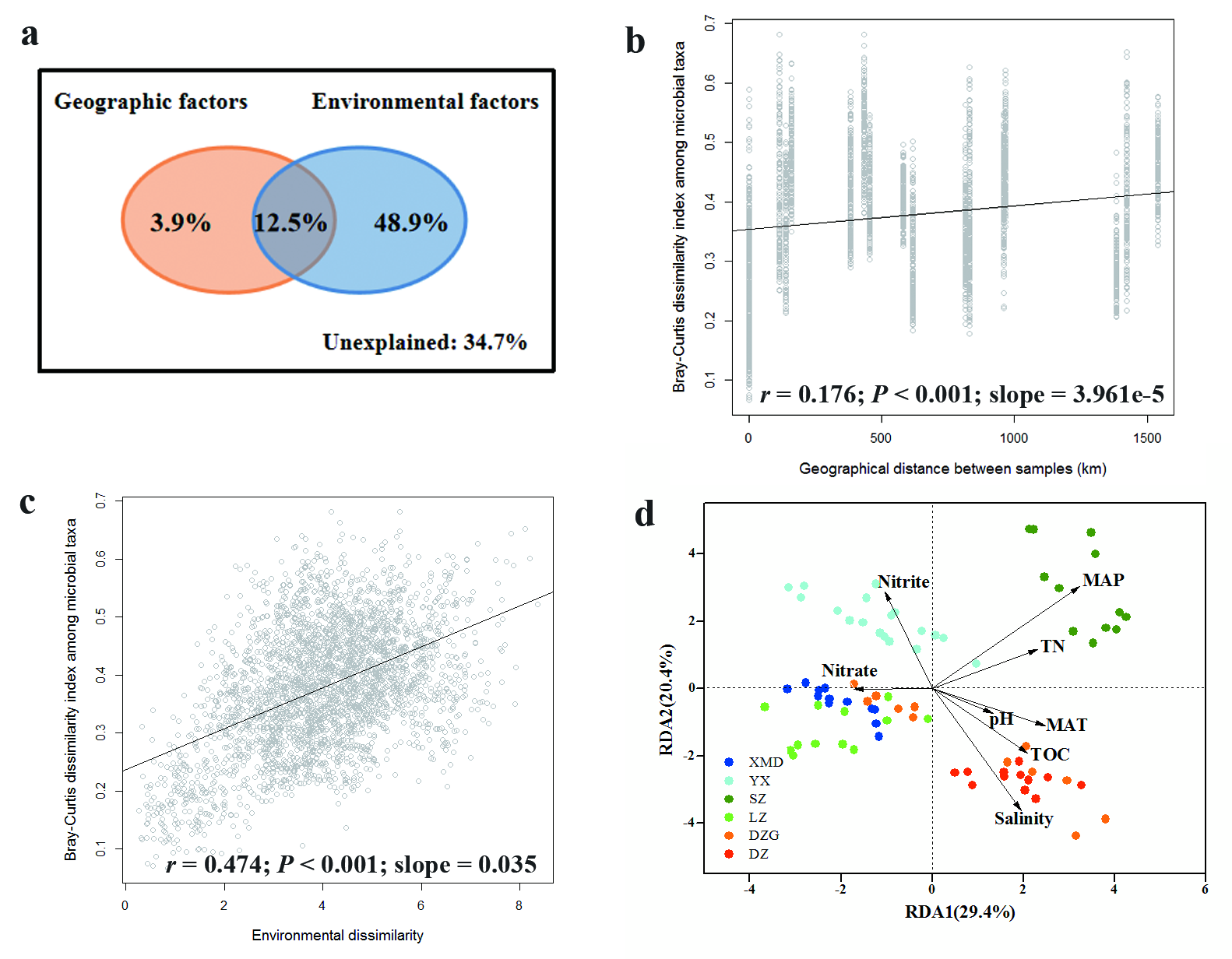

Supplement: FIG S6 [file mSystems.00442-19-sf006.tif]
