# Supplementary material for: Prokaryotic Diversity in Mangrove Sediments across Southeastern China Fundamentally Differs from That in Other Biomes
Source: mSystems. 2019 Sep 10;4(5):e00442-19. doi: 10.1128/mSystems.00442-19 (PMC6739103; doi:10.1128/mSystems.00442-19)
Supplement: TABLE S2 [file mSystems.00442-19-st002.docx]

**Table S2** Summary of results (F value and *P* value) of analysis of variance (ANOVA) showing the effects of site and plant type on dominant archaeal and bacterial phyla/classes. Values shown in bold are probability with significant results (*P* < 0.05).

|  | Site | | Plant type | | Site × plant type | |
| --- | --- | --- | --- | --- | --- | --- |
|  | F value | P value | F value | P value | F value | P value |
| Alphaproteobacteria | 37.249 | **<0.001** | 2.017 | 0.141 | 6.225 | **<0.001** |
| Betaproteobacteria | 22.124 | **<0.001** | 2.910 | 0.062 | 14.525 | **<0.001** |
| Deltaproteobacteria | 10.288 | **<0.001** | 10.150 | **<0.001** | 4.465 | **0.001** |
| Epsilonproteobacteria | 12.455 | **<0.001** | 6.121 | **0.004** | 12.100 | **<0.001** |
| Gammaproteobacteria | 35.379 | **<0.001** | 27.849 | **<0.001** | 7.474 | **<0.001** |
| Chloroflexi | 31.089 | **<0.001** | 23.697 | **<0.001** | 10.395 | **<0.001** |
| Bacteroidetes | 5.922 | **<0.001** | 1.624 | 0.205 | 2.389 | **0.047** |
| Planctomycetes | 28.616 | **<0.001** | 6.193 | **0.003** | 1.745 | 0.137 |
| Bathyarchaeota | 3.3851 | **0.009** | 5.2345 | **0.008** | 3.2274 | **0.012** |
| Acidobacteria | 43.236 | **<0.001** | 0.178 | 0.837 | 5.971 | **<0.001** |
| Euryarchaeota | 30.470 | **<0.001** | 2.231 | 0.116 | 2.704 | **0.028** |
| Gemmatimonadetes | 62.924 | **<0.001** | 3.264 | **0.045** | 5.573 | **<0.001** |
| Nitrospirae | 19.225 | **<0.001** | 9.947 | **<0.001** | 12.552 | **<0.001** |
| WS3 | 58.227 | **<0.001** | 1.948 | 0.151 | 6.793 | **<0.001** |
| Verrucomicrobia | 18.680 | **<0.001** | 5.463 | **0.006** | 7.532 | **<0.001** |
| Cyanobacteria | 3.413 | **0.008** | 5.838 | **0.005** | 1.283 | 0.282 |
